# Initial Findings From an Acute Hospital Care at Home Waiver Initiative

**DOI:** 10.1001/jamahealthforum.2023.3667

**Published:** 2023-11-03

**Authors:** Danielle Adams, Ashby J. Wolfe, Jessica Warren, Alexandre Laberge, Adam C. Richards, Kurt Herzer, Lee A. Fleisher

**Affiliations:** 1 Center for Clinical Standards and Quality, Centers for Medicare & Medicaid Services, Dallas, Texas; 2 Center for Clinical Standards and Quality, Centers for Medicare & Medicaid Services, Seattle, Washington; 3 Center for Clinical Standards and Quality, Centers for Medicare & Medicaid Services, Baltimore, Maryland; 4 Clinical Standards Group, Center for Clinical Standards and Quality, Centers for Medicare & Medicaid Services, Baltimore, Maryland

## Abstract

This cohort study assesses outcomes of patients treated during the initial 16 months of the Centers for Medicare & Medicaid Services Acute Hospital Care at Home initiative.

## Introduction

Hospital-at-home programs in the US have been limited to demonstration projects or small pilot programs for commercially insured patients. On November 25, 2020, as part of the Hospital Without Walls initiative to address the COVID-19 public health emergency and concerns about hospital bed capacity, the Centers for Medicare & Medicaid Services (CMS) launched the Acute Hospital Care at Home (AHCAH) initiative.^1,2,3^ Through a waiver-granting process, AHCAH allows individual CMS-approved hospitals to provide inpatient-level care in the home environment for Medicare fee-for-service and nonmanaged care Medicaid beneficiaries. CMS waived specific hospital Conditions of Participation (COP) that require 24-hour onsite nursing for patients and that patients’ homes meet certain structural and physical environment criteria.^4^ Participating hospitals must demonstrate their ability to meet the additional hospital COP that were not waived under section 1135 of the Social Security Act. This study reports on the cohort of patients treated during the initial 16 months of this initiative.

## Methods

This cohort study was covered by the Common Rule exemption described at 45 CFR 46.104(d)(4)(iv) and did not require institutional review board review. As of March 20, 2023, 277 hospitals across 123 systems in 37 states were approved to participate (eFigure, eTable 2 in Supplement 1). As part of the approval process, CMS performed an offsite review of hospital operations and processes to ensure that each hospital could satisfy unwaived COP in the home environment (Box). In addition to patient volume, hospitals agreed to self-report 2 data measures to CMS as a component of hospital participation. Patient escalation of care was defined as a patient’s return to the hospital to complete their course of care. Unexpected patient mortality was defined as a patient death while receiving acute inpatient care in the home, excluding those transferred to hospice (eTable 1 in Supplement 1). This study followed the relevant portions of the STROBE reporting guideline. No software was used for data analysis.

Box. **Selected Required Elements of the CMS Acute Hospital Care at Home Waiver^a^**

**Administrative Elements**

- Hospital point of contact for the waiver
- Hospital executive leader’s Attestation of Approval for waiver request submission
- Experience providing hospital care in the home

**Regulatory Elements**

- The provision of the following services as needed (either directly or under contract or arrangement):
  - Pharmacy
  - Infusion
  - Respiratory care including oxygen delivery
  - Diagnostic testing (eg, laboratory tests, imaging)
  - Monitoring with at least 2 sets of patient vital signs daily
  - Transportation to and from the hospital and the home
  - Food services including meal availability as needed by the patient
  - Durable medical equipment
  - Physical, occupational, and speech therapy
  - Social work and care coordination

**Safety Elements**

- At least 1 daily clinician visit (physician or advanced practice clinician), which can be remote after the initial in-person history and physical examination performed in the hospital or emergency department.
- At least 2 in-person daily visits by registered nurse or mobile integrated health practitioner or community paramedic. If both in-person visits are performed by mobile integrated health practitioner or community paramedic, additional daily remote registered nurse visit to develop a nursing plan.
- Immediate on-demand remote audio connection with an AHCAH team member who can immediately connect the appropriate registered nurse or physician.
- In-home appropriate emergency personnel response to a patient’s home within 30 minutes, if needed.
- Must develop or use patient selection criteria.
- Address advance care planning with patient prior to admission to the home.
- Implement a process for actions when a patient is unable to be reached within 15 minutes when arriving for a scheduled in-person or virtual visit.
- In-person registered nurse or mobile integrated health practitioner be present in the home to ensure that durable medical equipment is delivered and set up appropriately on the first home visit.

Abbreviations: AHCAH, Acute Hospital Care at Home; CMS, Centers for Medicare & Medicaid Services.

^a^
Not an exhaustive list of required elements.

## Results

A total of 11 159 patients were admitted under the waiver from November 25, 2021, through March 20, 2023, including 8417 with Medicare fee-for-service insurance, 1705 with nonmanaged care Medicaid insurance, and 1011 with both. The most common conditions treated, based on the primary diagnosis on the claim, are shown in the Table. For Medicare patients, the median length of stay obtained from claims was 5 days (IQR, 4-8). The overall proportion of patients transferred from home back to the hospital was 7.20%. During the study period, 38 unexpected deaths (0.34%) occurred in participating hospitals. Most unexpected deaths occurred in the setting of COVID-19 infection with the progression of more severe illness symptoms. With the exception of 3 cases, each of the patients who died had been transferred back to the hospital and received medical or intensive care unit–level care for several days prior to death.

**Table.** Most Common Diagnosis-Related Groups Treated Under Centers for Medicare & Medicaid Services Acute Hospital at Home Waiver in 8417 Fee-for-Service Medicare Patients

Diagnosis	Patients, No. (%)
Respiratory infections and inflammations with MCC	735 (8.7)
Heart failure and shock with MCC	425 (5.0)
Septicemia or severe sepsis without mechanical ventilation >96 h with MCC	257 (3.1)
Cellulitis without MCC	164 (1.9)
Simple pneumonia and pleurisy with MCC	158 (1.9)
Kidney and urinary tract infections without MCC	158 (1.9)
Chronic obstructive pulmonary disease with MCC	144 (1.7)
Septicemia or severe sepsis without mechanical ventilation >96 h without MCC	132 (1.6)
Simple pneumonia and pleurisy with CC	110 (1.3)
Respiratory infections and inflammations with CC	83 (1.0)

Abbreviations: CC, complication or comorbidity; MCC, major complication or comorbidity.

## Discussion

Patients who received care under AHCAH had a low mortality rate consistent with the hospital-at-home literature and minimal complications related to escalations back to the brick-and-mortar hospital. In addition to increased federal oversight, study limitations include that there may have been substantial selection bias from hospitals that chose to participate and patients who had medical conditions for which care could be provided in their homes.^5,6^ This pattern may be related to the safeguards inherent in the Medicare Hospital COP that have remained in place since the inception of AHCAH, appropriate patient selection, and the federal and local oversight incorporated into the initiative.

The CMS AHCAH initiative was extended through December 31, 2024, in the Consolidated Appropriations Act of 2023. This law extends waivers and flexibilities for physical environment requirements in the home, telehealth flexibilities, and the waiver that allows a hospital to provide routine services outside of the hospital. The law also requires hospitals to provide additional data to CMS to monitor the quality of care, and for CMS to undertake a comprehensive study of the AHCAH initiative by September 30, 2024. This study, data review, and additional monitoring will be important for identifying best practices that support safe and effective inpatient-level care delivered in the home environment.

## Notes

Supplement 1. **eMethods.** Supplemental Figure and Table **eFigure.** Geographic Distribution of the Use of the CMS Waiver as of March 20, 2023 **eTable 1.** Standard Operating Procedure for CMS Evaluation of Unexpected Mortality of a Patient Served by a Hospital With an Approved AHCAH waiver **eTable 2.** Attributes of a Successful Acute Hospital Care at Home Waiver (Participation) Request

Supplement 2.
Data Sharing Statement
