# Supplementary material for: Initial Findings From an Acute Hospital Care at Home Waiver Initiative
Source: JAMA Health Forum. 2023 Nov 3;4(11):e233667. doi: 10.1001/jamahealthforum.2023.3667 (PMC10625041; doi:10.1001/jamahealthforum.2023.3667)
Supplement: Supplement 1. — eMethods. Supplemental Figure and Table eFigure. Geographic Distribution of the Use of the CMS Waiver as of March 20, 2023 eTable 1. Standard Operating Procedure for CMS Evaluation of Unexpected Mortality of a Patient Served by a Hospital With an Approved AHCAH waiver eTable 2. Attributes of a Successful Acute Hospital Care at Home Waiver (Participation) Request [file jamahealthforum-e233667-s001.pdf]

Supplemental Online Content

Adams D, Wolfe AJ, Warren, J, Laberge A, Richards AC, Herzer K, Fleisher LA. Initial findings from an acute hospital care at home waiver initiative. *JAMA Health Forum.* 2023;4(11):e233667. doi:10.1001/jamahealthforum.2023.3667

eMethods. Supplemental Figure and Tables

eFigure. Geographic Distribution of the Use of the CMS Waiver as of March 20, 2023

eTable 1. Standard Operating Procedure for CMS Evaluation of Unexpected Mortality of a Patient Served by a Hospital With an Approved AHCAH Waiver

eTable 2. Attributes of a Successful Acute Hospital Care at Home Waiver (Participation) Request

This supplemental material has been provided by the authors to give readers additional information about their work.

© 2023 Adams D et al. *JAMA Health Forum*.

eMethods. Supplemental Figure and Tables

eFigure. Geographic distribution of the use of the CMS waiver as of March 20, 2023

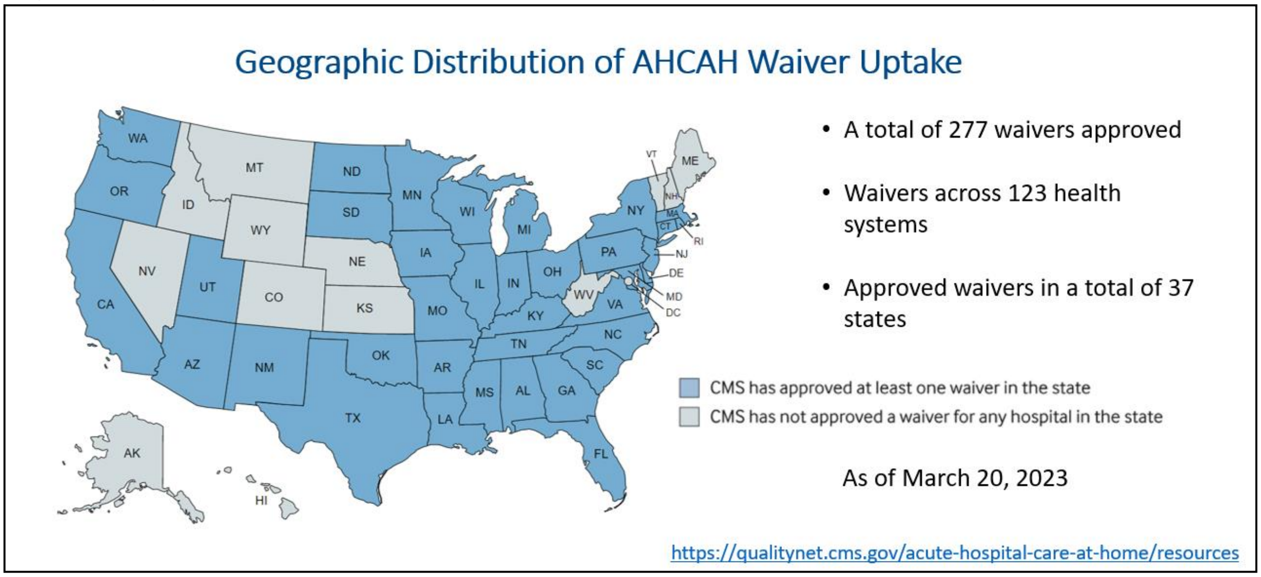

© 2023 Adams D et al. *JAMA Health Forum*.

eTable 1. Standard Operating Procedure for CMS Evaluation of Unexpected Mortality of a patient served by a hospital with an approved AHCAH waiver.

Unexpected Mortality Evaluation SOP

- **Critical alert for unexpected mortality received and reviewed by CMS team member within 24 hours**
    - Hospital POC contacted for supplemental information and details of the case
- **Supplemental information reviewed upon receipt;** call scheduled with hospital team to review details
- **Questions discussed during scheduled call with hospital**
    - What were the circumstances in which the patient was escalated back to the brick and mortar facility?
    - If the patient was not escalated back to the facility, what details informed the decision?
    - What was the response time from the initial change in condition to in-person contact with the patient?
    - How long did it take to get the patient back to the brick and mortar facility?
    - How did the program communicate with hospital staff about the return to the brick and mortar facility?
    - Did the hospital safety committee, or other workgroup, review the case?
    - What, if any, were the lessons learned by the program that could have affected the outcome?
- **CMS team reviews complete case information**
    - Identify key lessons learned and assess whether, overall, the HaH team
        - communicated well, and addressed changing needs in a timely fashion
        - appropriately escalated their patient (if applicable)
        - followed their established protocols
        - any concerns about this being an inappropriate admission to the HaH program
        - any concerns about the way the HaH team worked to honor the patient and family wishes during the course of care (if appropriate)

© 2023 Adams D et al. *JAMA Health Forum*.

eTable 2. Attributes of a successful Acute Hospital Care at Home waiver (participation) request.

Attributes of a Successful AHCAH Waiver Request

- **Standardize and socialize the team approach to care**
  - Clarify how the Hospital at Home team coordinates and continually communicates (daily huddles, interdisciplinary rounds, other)
  - Describe how the team supports a seamless patient transition to and from the home on day of admission, for routine diagnostics or urgent evaluation
- **Ensure the patient makes a clearly informed decision to participate in the Hospital at Home program**
  - Clearly review program expectations, possibility of escalation and details of program operations with patient and family or support person (if applicable)
  - Confirm code status, further clarify advance care planning desires; consider palliative care consultation if appropriate when patient admitted to HaH service
- **Develop a robust pharmacy workflow**
  - Leverage inpatient pharmacy expertise to coordinate ordering, dispensing, and delivery
  - Ensure real-time observation and documentation of any self-administration of medications
- **Identify clear protocols for oxygen and other DME delivery and function**
  - Demonstrate clear roles for medication administration among clinical staff
  - Designate a Hospital at Home care team member as the responsible party to confirm equipment is properly functioning, consistent with clinical orders
- **Coordinate with, and supervise, vendors and third party contractors**
  - Outline expectations of contracted entities to ensure clear expectations for performance, clinical documentation, and communication with the HaH team
  - Ensure clear lines of supervision which ultimately rest with hospital staff such as the Chief Nursing Officer
- **Ensure seamless data collection and communication**
  - Describe how critical diagnostic alerts are communicated to team members
  - Outline how vitals and other information gathered by technology integrates with the medical record
- **Develop a robust workflow for triage, evaluation and escalation**
  - Outline team capability for triage and response by HaH care team members at all hours of day or night
  - Maintain a standardized protocol for communication between EMS/911, HaH team members and receiving facility staff in the event of an escalation
  - Confirm ongoing communication and coordination with local EMS (as applicable) to ensure patients are appropriately identified and brought to the hospital if escalation is necessary
  - Familiarize patient and family with care expectations including possibility of escalation should clinical condition change

© 2023 Adams D et al. *JAMA Health Forum*.
