# Supplementary material for: Initial Findings From an Acute Hospital Care at Home Waiver Initiative
Source: JAMA Health Forum. 2023 Nov 3;4(11):e233667. doi: 10.1001/jamahealthforum.2023.3667 (PMC10625041; doi:10.1001/jamahealthforum.2023.3667)
Supplement: Supplement 2. — Data Sharing Statement [file jamahealthforum-e233667-s002.pdf]

Data Sharing Statement

Adams. Initial Findings From an Acute Hospital Care at Home Waiver Initiative. *JAMA Health Forum*. Published November 03, 2023. doi:10.1001/jamahealthforum.2023.3667

Data
Data available: Yes
Data types: Deidentified participant data, Data dictionary
How to access data: The data will be available upon publication in the RESDAC
When available: With publication

Supporting Documents
Document types: None

Additional Information
Who can access the data: Researchers who submit a request
Types of analyses: For research purposes
Mechanisms of data availability: Through application to the RESDAC and approval
